# A Case of Onanism in a Boy Seven Years Old

**Published:** 1854-02

**Authors:** A. Garwood

**Affiliations:** Cassapolis


*A case of Onanism in a boy seven years old.* By A. Garwood, M. D.

The following case of onanism is so unusual and remarkable, at least so far as my experience and information extends, that I thought it might prove interesting to the readers of your journal.

The patient was a boy, I took out of the county poor house, to live with me and had him bound by the superintendents of the county poor, till he was twenty-one years of age.

He was seven years old, very fair complexion, light hair, black eyes, a slender delicate frame, and apparently an innocent, sprightly and interesting child.

I did not suspect him of being a masturbate, till I caught him in the act of self pollution. I then learned from him that he was taught the loathsome practice at school when but four years of age; and that the habit had become confirmed and had been growing upon him ever since. I punished him at the time, and gave him a lecture on the consequences of the habit if continued. Told him that it would injure his health and mind, that it would make him a weakly, foolish, good for nothing boy, that other children would not be allowed to play with him, and that I would take him back to the Poor House. He seemed very penitent and promised reformation.

Never having had much experience in such cases, I thought the means I had used might possibly cause him to discontinue the filthy practice. But I soon learned that he did not quit it for a single day or night. He commenced living with me in the summer, and the habit grew upon him during the fall and winter rapidly, as was evident from the stains on his linen, from his general appearance, and from his own confession. When he found, that he could not conceal the fact from me any longer, he became very bold about it, and seemed to loose all shame and delicacy of feeling on the subject. He stated that he never missed a night but that he indulged in it two or three times, that he engaged in it at the privy, that when he went to school, instead of playing with the other boys, he would sneak off by himself to practice it, and whenever he could get off by himself at any time or place, he was at it. He now declared that he could not, and would not quit it, because "he was so used to it." I could not extort a promise from him to quit it, and he concluded that he would rather go back to the Poor House, than to leave off the practice. The symptoms at this time were emaciation, inactivity, did not want to play, but would set for hours listless and heedless of what was going on—his mind seemed more dull about everything, except the gratification of his passion, for which in seeking opportunities he showed great acuteness and deception. He was very stiff in his limbs and back, so that it required quite an effort for him to exercise. There was a dark areola beneath the eyes. He could not look a person in the face, had an excellent appetite, ate hearty, and craved the heartiest kind of food—not having missed a meal during the seven months he lived with me. When kept from it through the day by close watching, he became almost frantic, he would thrust his finger in his nostril, often making it bleed, would rub between the fingers of one hand with the fore-fingers of the other, and seemed to be perfectly on nettles, as though he could hardly endure it.

But the most prominent and disgusting symptom of all was incontinence of urine. He lost the control of the sphincter of the bladder, to such an extent, that immediately after indulging, he had to urinate several times, and often kept his clothes saturated half the time, in consequence of being unable to retain his urine, till he could get to a proper place to evacuate. He has a great many times wet his pants at the table, and often had to leave it in the middle of a meal to run to the privy, and very often failed to get there in time.

*Treat.*—After using every moral means in my power, I tried cold bathing, restricting his diet to plain unstimulating food, whipping him as hard as I dared to without injuring the child, blistered his penis till it was all over raw, and as a *dernier resort* tied his hands. All these efforts were entirely abortive; whilst his penis was raw, he indulged as much as ever, and did not seem to regard the soreness. And when his hands were tied, he would bring on a seminal discharge by friction against his clothes, between his thighs, or between his abdomen and bed clothes, and at last he obtained such command over the abdominal, perineal and glutial muscles, in connection with the force of imagination, that he could produce a discharge sitting on a chair in my presence when there was no motion perceptible. The desire of self gratification appeared to be constantly in his mind, and I am convinced that he would forgo any and everything else, even death itself, before he would quit the practice. Giving up all hopes of effecting a cure, and his presence becoming so disgusting and repulsive, I laid the case before the superintendents of the county and the board of supervisors, accompanied with the request, that they would destroy the indentures, and receive him again as a pauper, which they did accordingly.

What's to be done with such a case? Would it be justifiable to emasculate under such circumstances? If that did unman him, it might prevent him teaching others the same trick, (for he says he has made a business of teaching others whenever he had an opportunity.)

The above case should teach parents and guardians, to exercise a watchful care over those under their charge, in reference to this loathsome habit, and also the almost unpardonable nature of the crime of older persons teaching the younger or uninitiated this vile practice.

Cassapolis, January 15th, 1854.

